# Sensing behavior of flower-shaped MoS_2_ nanoflakes: case study with methanol and xylene

**DOI:** 10.3762/bjnano.9.57

**Published:** 2018-02-16

**Authors:** Maryam Barzegar, Masoud Berahman, Azam Iraji zad

**Affiliations:** 1 Nanotechnology Research Institute, Sharif University of Technology, Tehran, Iran; 2 Physics Department, Sharif University of Technology, Tehran, Iran

**Keywords:** density functional theory, gas sensor, hydrothermal method, methanol, MoS_2_ nanoflakes, xylene vapor

## Abstract

Recent research interest in two-dimensional (2D) materials has led to an emerging new group of materials known as transition metal dichalcogenides (TMDs), which have significant electrical, optical, and transport properties. MoS_2_ is one of the well-known 2D materials in this group, which is a semiconductor with controllable band gap based on its structure. The hydrothermal process is known as one of the scalable methods to synthesize MoS_2_ nanostructures. In this study, the gas sensing properties of flower-shaped MoS_2_ nanoflakes, which were prepared from molybdenum trioxide (MoO_3_) by a facile hydrothermal method, have been studied. Material characterization was performed using X-ray diffraction, Brunauer–Emmett–Teller surface area measurements, elemental analysis using energy dispersive X-ray spectroscopy, and field-emission scanning electron microscopy. The gas sensing characteristics were evaluated under exposure to various concentrations of xylene and methanol vapors. The results reveal higher sensitivity and shorter response times for methanol at temperatures below 200 °C toward 200 to 400 ppm gas concentrations. The sensing mechanisms for both gases are discussed based on simulation results using density functional theory and charge transfer.

## Introduction

Recent efforts in exploring two-dimensional (2D) materials have led to the introduction of a new family of materials known as transition metal dichalcogenides (TMDs), which show remarkable electrical, optical and transport properties [1–13]. TMDs are a group of materials with the general formula MX_2_, where M is a transition metal element of group IV, V or VI, and X is a chalcogen (S, Se, or Te) [1–13]. Their properties, including a large surface-to-volume ratio, high process compatibility and flexibility, make them good candidates for sensing applications [8,14–15]. One of the most explored materials in this group is MoS_2_, which is a semiconductor with a variable band gap based on the number of layers [14–15]. Numerous methods have been applied to synthesize single or few-layered MoS_2_, including but not limited to mechanical cleavage, chemical exfoliation, hydrothermal synthesis and chemical vapor deposition [16–23]. The hydrothermal process is a scalable method to synthesize MoS_2_ nanosheets and nanoflakes. There are numbers of articles which report the successful growth of flower-like MoS_2_ nanoflakes using this technique [19–23]. Due to the high surface-to-volume ratio, activity, tunable band gap, low electrical noise and acceptable electrical conductivity, MoS_2_ is considered as one of the most suitable candidates to use in gas sensing devices [14–15].

There are few reports on gas sensing properties of MoS_2_. Cantalini et al. [8] reported the response of few layer MoS_2_ films to NO_2_ at sub-ppm concentrations and reasonable sensitivity to 1 ppm NO_2_ with fast and reversible response at 100 °C. It has been shown that charge transfer between MoS_2_ and NO_2_ or NH_3_ molecules can be considered as the main reason behind the changes in resistance [24]. In another report, the remarkable potential of MoS_2_ in sensing triethylamine molecules has been investigated. It has been shown that MoS_2_ is a good sensor to detect acetone with response to methanol [14]. There are also investigations that marked the high response of MoS_2_ toward methanol and ethanol [15]. Besides these experimental reports, there are numerous theoretical investigations which illustrate the potential of MoS_2_ for detecting various gas molecules [25–26]. Among these reports, the properties of flower-shaped MoS_2_ as a gas sensor is underestimated, hence in this paper, we demonstrate this potential. The flower-shaped MoS_2_ can easily be grown using the inexpensive hydrothermal technique with high quality and in large quantity, which reduces the final cost of the sensor. To demonstrate this potential, we consider xylene and methanol molecules as the target gases.

Xylene is a nonpolar colorless flammable gas that not only pollutes the environment but is also directly harmful to human health as a carcinogenic gas. Since xylene exists in the mixture of gasoline, in the solvent components of commonly used commercial products, and in some paint and varnishes, monitoring the existence of this gas in the related industries is of vital importance [27–30]. Methanol, on the other hand, is a polar, colorless, flammable molecule which is highly toxic for human both in liquid and vapor form. The inhalation of methanol vapor may cause serious problems for metabolism, which highlights the importance of monitoring this gas in the environment [27–30].

In this paper, we explore the gas sensing potential of pure flower-shaped MoS_2_ nanoflakes toward xylene and methanol vapors. We show that these flower-shaped MoS_2_ nanoflakes have adequate surface-to-volume ratio and active surface sites to detect gas molecules and our results reveal that they can detect methanol and xylene with good response.

This paper is organized as follows: in the Experimental and Simulation section, details of the growth and the setup of our research are discussed. In addition, the resulting MoS_2_ nanoflakes are characterized. The simulation process is also discussed in this section. In the Results and Discussion section, our results on gas sensing properties of the produced MoS_2_ toward xylene and methanol are presented. In addition, the selectivity of our device toward various available gas molecules and the mechanism of adsorption as well as the simulation results are discussed.

## Experimental and Simulation Data

### Synthesis of MoS_2_ few-layer nanoflakes

Among various methods for preparing MoS_2_ few-layer sheets, we followed the synthesis process reported before in [23]. In short, the MoS_2_ sheets were synthesized through sulfurization of MoO_3_ powder in an aqueous medium as follows: 0.05 g MoO_3_ powder and 0.13 g thiourea were dissolved in 40 mL deionized water followed by rapid stirring for about 30 min. Subsequently, the mixture was transferred into a 50 mL teflon-lined stainless steel autoclave and maintained at 200 °C for 24 h. After cooling naturally, the black MoS_2_ product was collected by filtration, washed with distilled water and pure ethanol for several times and then dried under vacuum at 45 °C overnight.

### Characterization

X-ray diffraction (XRD) patterns were obtained with an X-ray diffractometer (Shimadzu XRD-6000, Cu Kα radiation) and energy dispersive X-ray (EDX) spectra and sample morphology were characterized by field-emission scanning electron microscopy (FE-SEM, TE-SCAN, MIRA3). The Brunauer–Emmett–Teller (BET) surface area of the products was analyzed using a Micromeritics nitrogen adsorption apparatus.

### Fabrication of gas sensors

An alumina wafer with an area of 1 cm^2^ was considered as the proper substrate for our gas sensors. The substrates were immersed in acetone and isopropyl alcohol and sonicated for 15 min to remove any undesired surface ions. A 200 nm Pt film as a snake-shaped heater was sputtered on the back of the substrate using a sputter coater from Nanostructured Coatings Co. To fabricate the sensor device, dispersed flower-shaped MoS_2_ nanoflakes in ethanol were first deposited on a 1 × 1 cm^2^ alumina wafer by spin coating. Then the gold microelectrodes were deposited on selective areas of the prepared, uniform, thin film through a comb-shaped shadow mask by sputtering. The mask was then lifted off and the microelectrodes with 100 µm width and a 200 µm gap between each electrode remained and were annealed at 200 °C for 120 min for better film adhesion.

### Gas sensing measurements

In order to test the samples toward different gas molecules, a dynamic system based on N_2_ as a carrier gas is used. For testing the sensor operation, the target vapors were produced by bubbling dry air through the respective solvents. The amount of xylene and methanol as target gases were controlled by two mass flow controllers. The concentration of xylene and methanol was calculated using Equation 1 [30]:

[1]
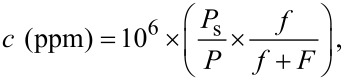


where *f* and *F* are the flow rates (in sccm) of the bubbling N_2_ saturated with the vapors and the N_2_ gas, respectively; *P* is the total pressure and *P*_s_ is the saturated vapor partial pressure obtained by the Antoine equation [31]. Special care with regards to temperature control was taken so that the formula is valid in our system throughout the testing period.

The chemoresistance response is defined as (*R*_air_ – *R*_gas_)/*R*_air_ where *R*_air_ and *R*_gas_ are the resistance in N_2_ and the mixed gases in different concentrations, respectively.

### Simulation

A super-cell containing 25 primitive unit cells of monolayer MoS_2_ was considered as the pristine model. Then, a sulfur atom at the center of the model was removed and fully relaxed to study the sulfur vacancy. After that, the detection of xylene and methanol with these models were studied. Density functional theory in the local-density approximation with a Perdew–Zunger correlation function was considered in a double-zetta polarized scheme with a mesh cut-off sampling of 75 Ry for optimization and study [32]. The Monkhorst–Pack mesh with 21 × 21 × 1 sampling was used for the investigation. The optimization process continued until the force on each atom was lower than 0.01 eV. The Grimme-DFT-D2 method was used to model the van der Waals interactions that may occur in the simulation. All calculations are performed using the well-known siesta package [33–34].

## Results and Discussion

**Figure 1:**
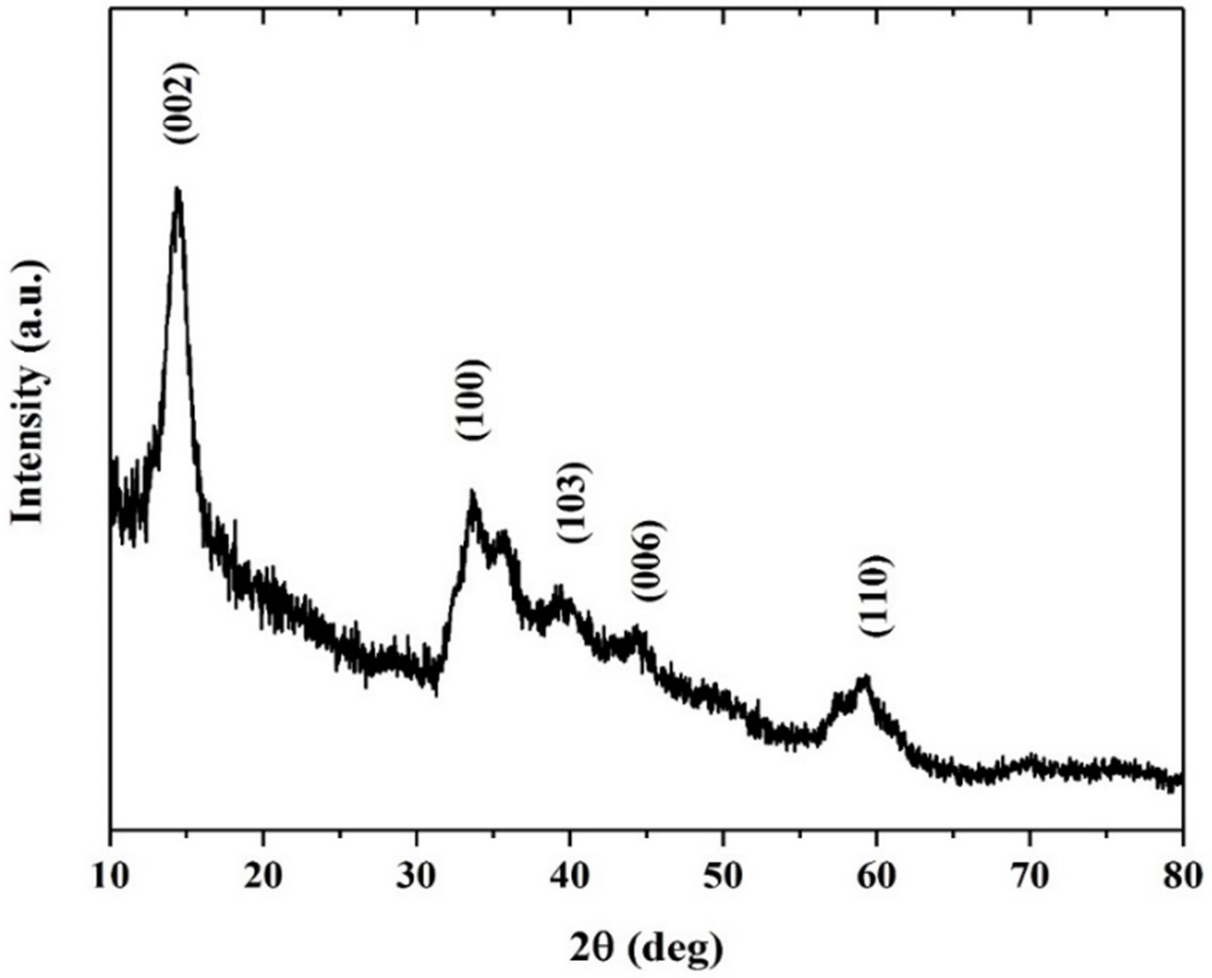
The X-ray diffraction pattern of flower-shaped MoS_2_ nanoflakes synthesized by the hydrothermal method.

The crystalline structure and phase purity of the dried black MoS_2_ powder were investigated using XRD. As illustrated in Figure 1, there are two completely distinguishable peaks at 33.61° and 59.19° which are in good agreement with corresponding peaks of the (100) and (110) planes of MoS_2_ sheets with hexagonal crystal structure denoted as the 2H phase (JCPDS-37-1492). The measured *d*-spacings of 0.26 nm and 0.15 nm are related to the (100) and (110) planes, respectively. The broadening of the XRD peaks may indicate nanometer-size growth of MoS_2_ with few-layer stacks.

**Figure 2:**
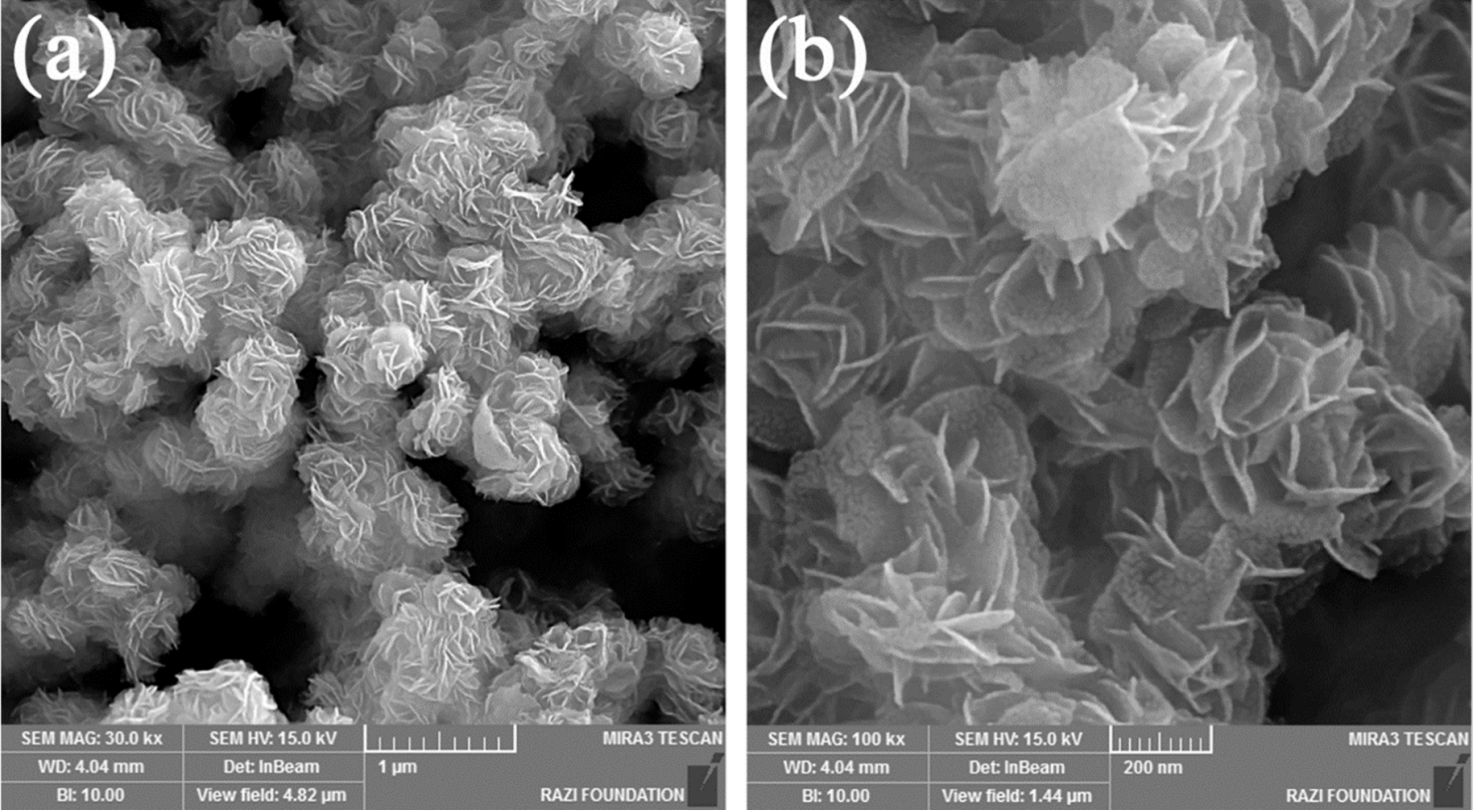
FE-SEM micrographs of flower-shaped MoS_2_ nanoflakes synthesized via the hydrothermal method.

In order to confirm such results, the size and morphology of the prepared MoS_2_ samples were observed using FE-SEM as shown in Figure 2a, and in higher magnification in Figure 2b. As shown in the micrographs, flower-like particles are grown from few stacking nanoflakes with almost 20 nm thickness. To further investigate the quality of these growth structures, EDX data were obtained.

**Figure 3:**
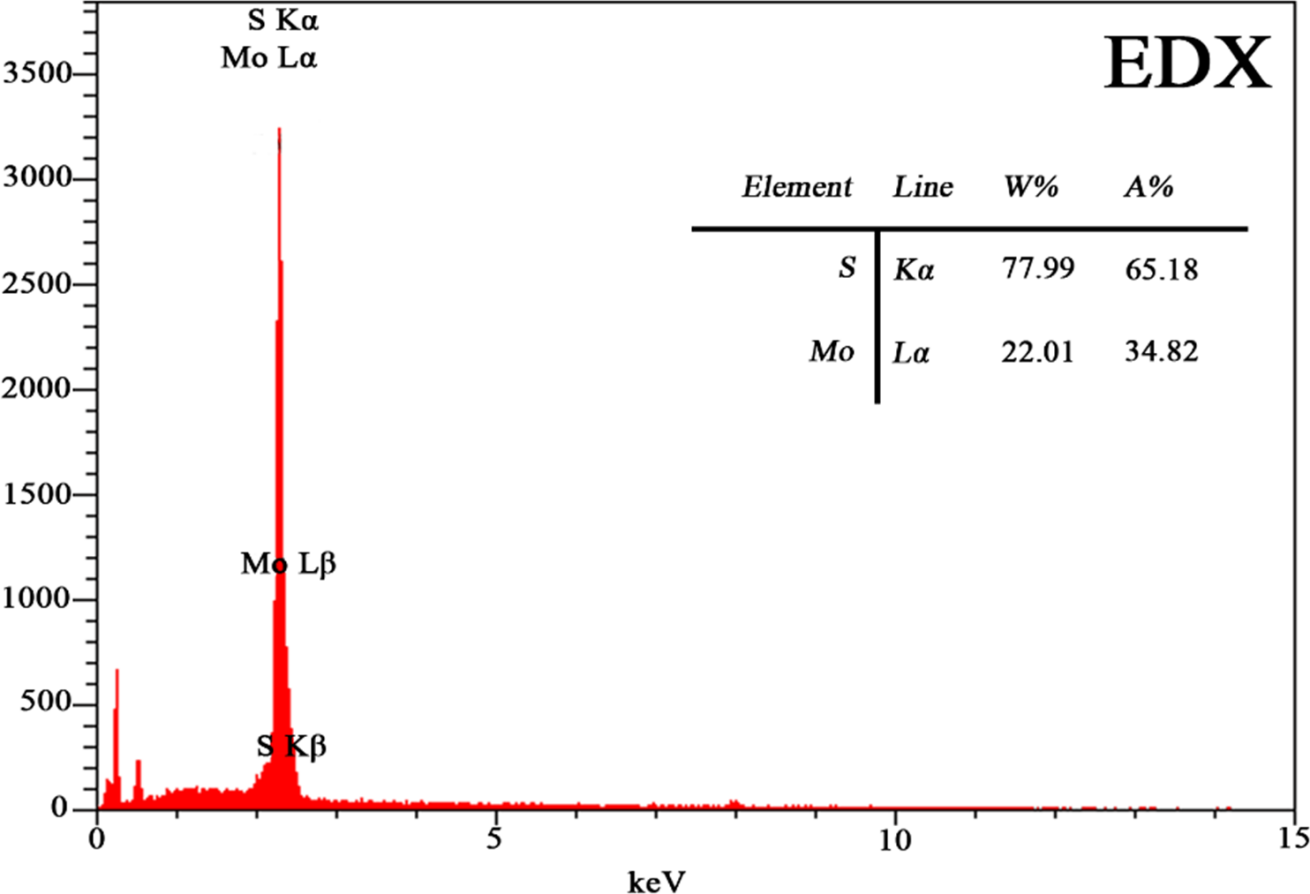
EDX analysis of MoS_2_ nanoflake powder as prepared by the aqueous hydrothermal method.

Figure 3 shows an S to Mo atomic ratio of about 1.87, indicating sulfur vacancies in the samples. The previous report shows that sulfur vacancies can increase the possibility of charge transfer in MoS_2_ nanoflakes which may act as the main reason to alter the conductivity [35]. It has been also reported that the crystal phase and edge play a significant role in the electro-activity of MoS_2_ nanosheets. Furthermore, sulfur vacancies contribute significantly to the electronic properties of MoS_2_ [36–37]. Hence, such sulfur vacancy is desirable for the gas sensing properties of MoS_2_.

To study the application of the flower-shaped MoS_2_ for gas sensing, the Brunauer–Emmett–Teller (BET) experiment has been performed. The measured surface area is 64.14 m^2^ g^−1^, which is more than two times higher compared with the bulk MoS_2_ (average 27 m^2^ g^−1^) [38].

**Figure 4:**
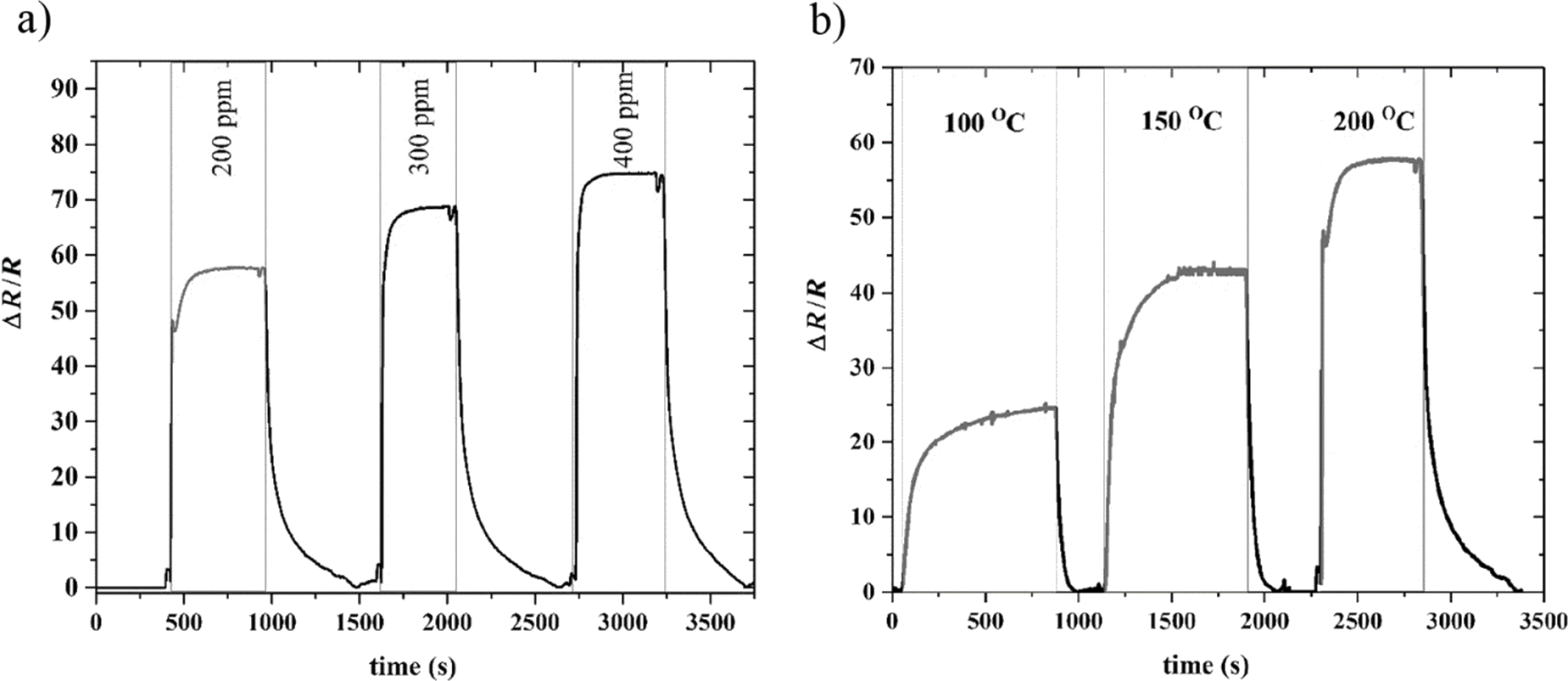
Typical response of MoS_2_ nanoflakes toward methanol for: (a) different concentrations at 200 °C working temperature, (b) for 200 ppm at different working temperatures.

Figure 4 shows the dynamic response of the fabricated sensor to different concentrations of methanol vapor at working temperatures in the range of 100 to 200 °C. A very small response towards target gases was observed at a temperature below 100 °C. As the sensor is exposed to concentrations of methanol in the range of the health exposure limits (i.e., 200–400 ppm), it reacts quickly and the resistance reduces. Figure 4a clearly illustrates the reversible behavior of about 120 s and 370 s as the response and recovery times for 200 ppm, respectively. When the working temperature was increased, the sensitivity was improved from 25 to 55 for 200 °C and the response time decreased from about 800 s to 120 s (Figure 4b).

**Figure 5:**
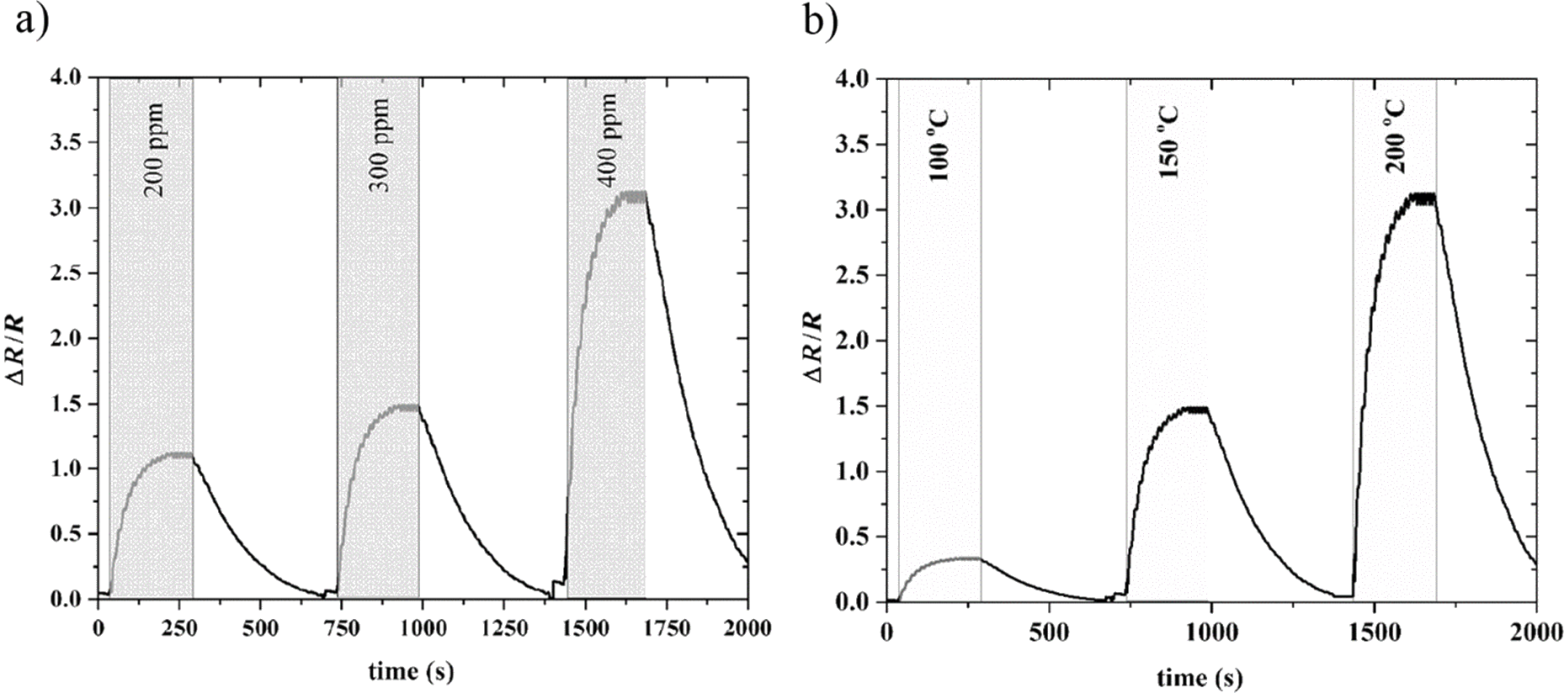
Typical response of MoS_2_ nanoflakes toward xylene for: (a) different concentrations at 200 °C working temperature, (b) for 400 ppm at different working temperatures.

In addition, we studied the sensing ability of the flower-shaped MoS_2_ nanosheets toward xylene. Figure 5a,b represents the gas sensing response toward 200–400 ppm of xylene vapor at the temperature range from 100 to 200 °C. When the temperature was increased, the sensitivity was enhanced from about 0.25 to almost 3 for 400 ppm xylene, while the response and recovery time decreased from about 250 and 500 s to 150 and 450 s, respectively. As illustrated in Figure 5a, when increasing the concentration at 200 °C from 200 to 400 ppm, the sensitivity was improved from 1 to 3, which represents the potential of the fabricated sensor in detecting different concentrations of xylene.

**Figure 6:**
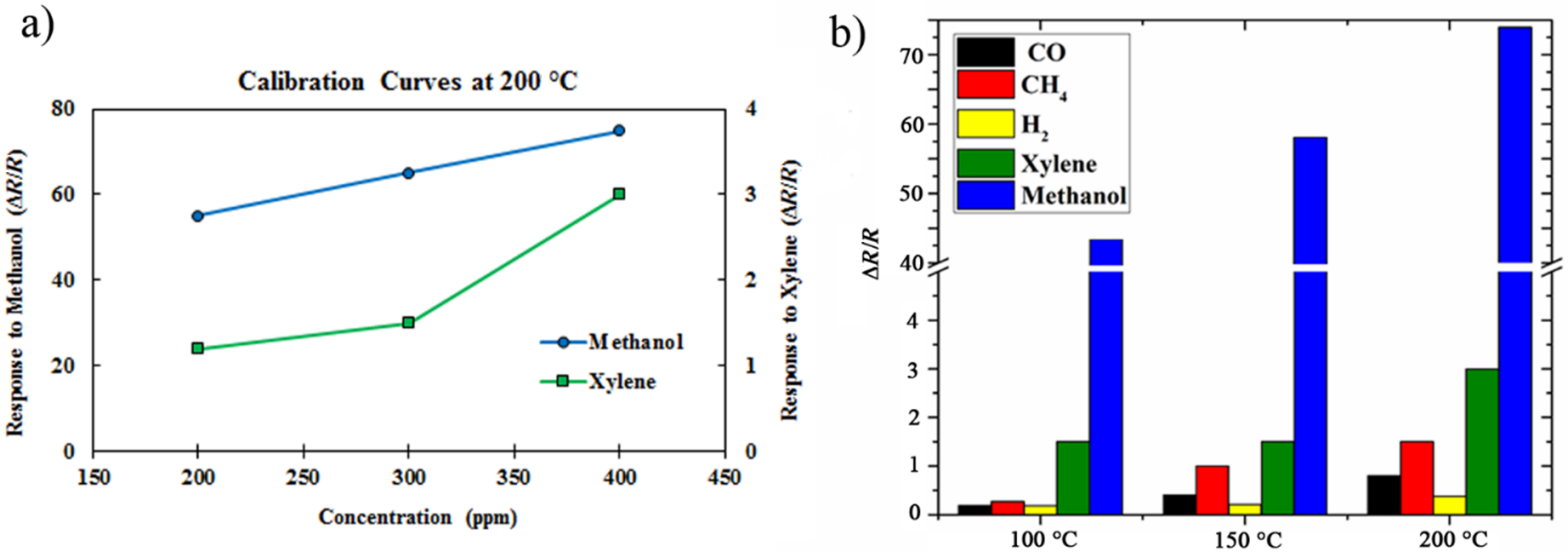
**(**a) Calibration curves of flower-shaped MoS_2_ nanoflakes towards xylene and methanol at 200 °C; (b) the sensitivity of the samples towards 400 ppm of CO, CH_4_, H_2_, xylene, and methanol at different working temperatures.

As indicated in Figure 6a, it is clear that the sensor responds to both methanol and xylene gases, while the former is more linear with higher sensitivity. In order to study the selectivity of our sensor toward available gases in the laboratory, the response toward 400 ppm concentration of H_2_, CH_4_ and CO has also been studied. Figure 6b indicates that the sensor is less sensitive than methanol at all working temperatures. It is worth mentioning that when increasing the temperature up to 200 °C, the sensitivity of our samples increased. This response enhancement can be interpreted as methanol decomposition to syngas (CO/H_2_) at around 433 K [39], and ethanol formation from the syngas due to the catalytic properties of MoS_2_ at around 185 °C [40]. It is known that ethanol has a higher electron-donor rate than methanol [41]. Hence, a larger variation in sample resistance at higher temperatures can be expected.

To study the reason behind this repeatable behavior toward both gases, we consider the flower-shaped MoS_2_ as an n-type semiconductor due to its bulk shape, which was also confirmed using Hall effect experiments. Four Ti (10 nm)/Au (200 nm) films as electrodes in van der Pauw configuration were deposited on spin-coated MoS_2_/SiO_2_ (300 nm)/Si samples for Hall effect measurements. The details of the configuration are given in [42]. The results show an n-type characteristic for the samples with an electron density and a mobility of about 6.3 × 10^13^ cm^−3^ and 75 cm^2^ V^−1^ s^−1^, respectively. When xylene vapor (as an electron donor) is exposed to the sensing device, it reduces the resistance of the device due an increase in the majority carrier concentration. The same process can be seen for methanol, acting as powerful electron-donor gas molecules. This increment in the electron carrier concentration can reduce the resistivity of the device. When the gas leaves the chamber, the molecules which transfer the charge from weak van der Waals interaction leave the MoS_2_ layer and the concentration of the carrier reduces, hence the resistance returns back to its initial value. In the hydrothermal growth of MoS_2_ (as proven before), the high edges, corners and vacancies provide proper sites for reaction and sensing of the gas molecules [43].

**Figure 7:**
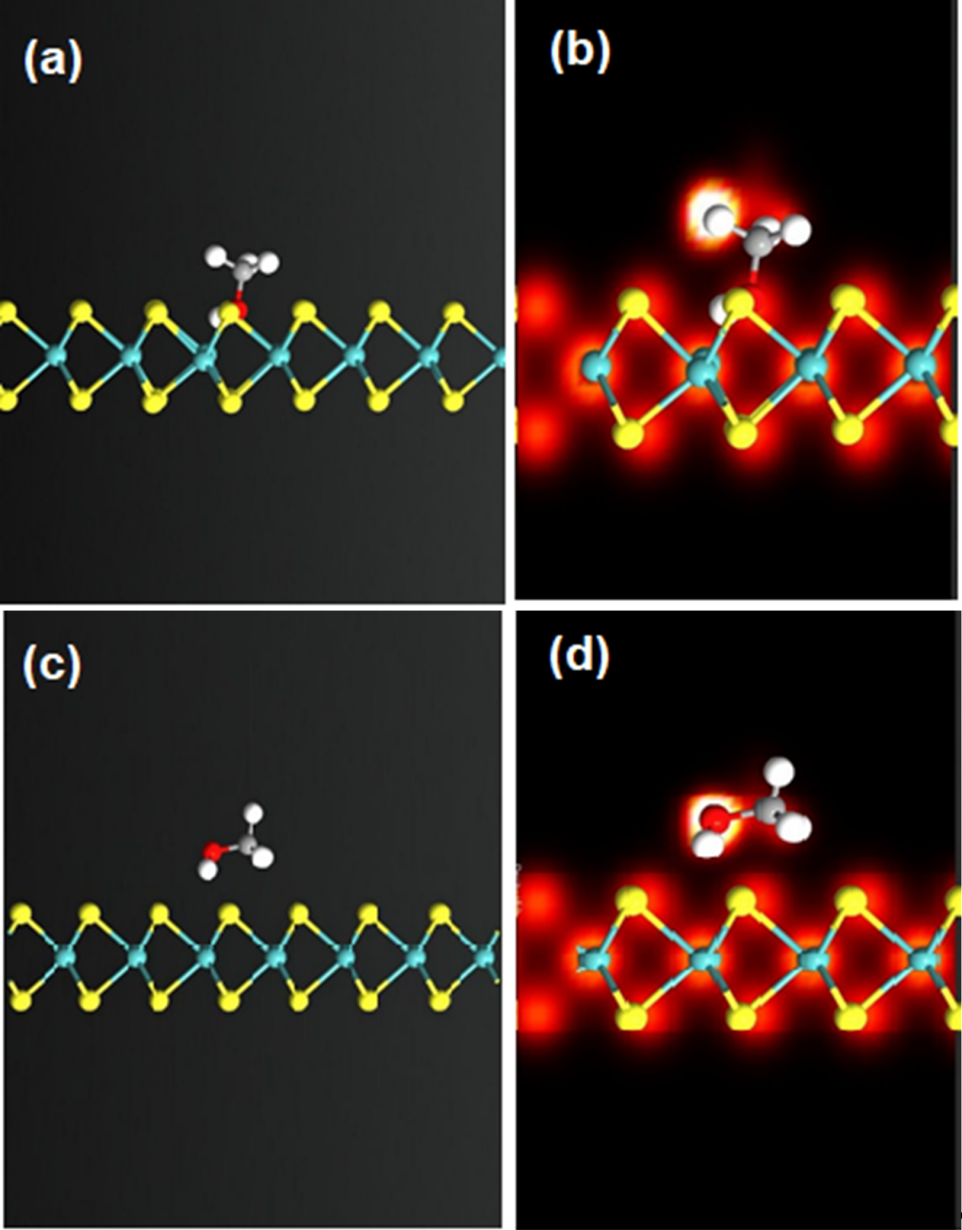
The optimized structure of methanol on (a) defect-containing MoS_2_. (b) The corresponding electron density of methanol on defect-containing MoS_2_. (c) The relaxed structure of methanol on pristine MoS_2_. (d) The electron density of methanol on pristine MoS_2_.

A scrutinized investigation of the results revealed that when methanol enters the chamber, the sensor shows a fast response which is followed by a slow one, while the sensor shows only a slow process toward xylene. It may be related to the existence of one reaction mechanism for xylene as a nonpolar molecule and two distinct mechanisms for methanol as a polar one. To find such differences, we performed a simulation study on the pristine monolayer and similar surface with sulfur vacancy of MoS_2_ in the presence of xylene and methanol molecules. Xylene shows almost no interaction with pristine MoS_2_ while a van der Waals interaction happens in the case of sulfur vacancy, which leads to charge transfer from xylene to MoS_2_. Since only defect sites can aquire charges from xylene, we expect only one mechanism for gas detection of this kind. Figure 7 illustrates the interaction of methanol on pristine and sulfur-vacancy MoS_2_ and the electron density of these materials. As can be seen, methanol can transfer charge via van der Waals interactions both toward the pristine and the defect-containing MoS_2_. These two different detection sites for methanol can result in two distinct mechanisms that are observed in our experiments. The fast process is related to charge transfer on the pristine sites while the slow one may be related to the physical adsorption of the methanol molecules on the deficient sites. It can be assumed that the sulfur vacancies can provide active sites for gas molecules to interact with MoS_2_ as well as altering the position of sub-bands in the band structure [44].

The Mulliken population shows that the charge transfer in the case of xylene on pristine and the defect-containing MoS_2_ is almost 0.001e and 0.027e (average of different isomers), respectively, in the area of study. In the case of methanol, the Mulliken population is 0.04e and 0.071e in the pristine and the defect sites, respectively. These results demonstrate that both xylene and methanol transfer a fraction of a charge to MoS_2_, which lead to an increase in carrier concentration and reduction of sample resistance.

## Conclusion

In summary, we demonstrated a facile and efficient hydrothermal synthesis of MoS_2_ nanoflakes. It was found that the nanoflakes form a flower-shaped structure with a large surface-to-volume ratio. The sensor device was successfully fabricated by spin coating of MoS_2_ flakes on an alumina substrate, on the back side of which a Pt heater circuit for thermal annealing was deposited. Our results indicate that the produced, flower-shaped MoS_2_ nanoflakes showed sensitivity towards methanol and xylene as polar and nonpolar gas molecules. We discussed changes in resistivity toward gas molecules according to a charge transfer mechanism. It was found that both gases can be detected in concentrations as low as 200 ppm while the detection sensitivity increased with increasing gas concentration. It was also confirmed that the response and recovery time for sensors decreased dramatically with increasing temperature. The sensitivity of the produced sensor device towards methanol was found to be higher than for xylene, as the calibration curve indicated a linear response to increasing concentration. In addition, the simulation results showed that xylene interacts with defect sites while methanol can interact with both the pristine and the defect sites, leading to higher sensitivity, which coincides well with the experimental results of this study.
